# PSMA-Expression Is Highly Associated with Histological Subtypes of Renal Cell Carcinoma: Potential Implications for Theranostic Approaches

**DOI:** 10.3390/biomedicines11113095

**Published:** 2023-11-20

**Authors:** Vinh Ngoc Bui, Lena M. Unterrainer, Matthias Brendel, Sophie C. Kunte, Adrien Holzgreve, Fabian Allmendinger, Peter Bartenstein, Frederick Klauschen, Marcus Unterrainer, Michael Staehler, Stephan Ledderose

**Affiliations:** 1 Department of Nuclear Medicine, LMU University Hospital, LMU Munich, 81377 Munich, Germany; matthias.brendel@med.uni-muenchen.de (M.B.); sophie.kunte@med.uni-muenchen.de (S.C.K.); fabian.allmendinger@gmx.de (F.A.); peter.bartenstein@med.uni-muenchen.de (P.B.); marcus.unterrainer@med.uni-muenchen.de (M.U.); 2 Munich Cluster for Systems Neurology (SyNergy), 81377 Munich, Germany; 3 Institute of Pathology, LMU Munich, 81377 Munich, Germany; frederick.klauschen@med.uni-muenchen.de (F.K.); stephan.ledderose@med.uni-muenchen.de (S.L.); 4 Die RADIOLOGIE, 80331 Munich, Germany; 5 Department of Urology, LMU University Hospital, LMU Munich, 81377 Munich, Germany; michael.staehler@med.uni-muenchen.de

**Keywords:** PSMA, renal cell carcinoma, theranostics, immunohistochemistry

## Abstract

In renal cell carcinoma (RCC), accurate imaging methods are required for treatment planning and response assessment to therapy. In addition, there is an urgent need for new therapeutic options, especially in metastatic RCC. One way to combine diagnostics and therapy in a so-called theranostic approach is the use of radioligands directed against surface antigens. For instance, radioligands against prostate-specific membrane antigen (PSMA) have already been successfully used for diagnosis and radionuclide therapy of metastatic prostate cancer. Recent studies have demonstrated that PSMA is expressed not only in prostate cancer but also in the neovasculature of several solid tumors, which has raised hopes to use PSMA-guided theranostic approaches in other tumor entities, too. However, data on PSMA expression in different histopathological subtypes of RCC are sparse. Because a better understanding of PSMA expression in RCC is critical to assess which patients would benefit most from theranostic approaches using PSMA-targeted ligands, we investigated the expression pattern of PSMA in different subtypes of RCC on protein level. Immunohistochemical staining for PSMA was performed on formalin-fixed, paraffin-embedded archival material of major different histological subtypes of RCC (clear cell RCC (ccRCC)), papillary RCC (pRCC) and chromophobe RCC (cpRCC). The extent and intensity of PSMA staining were scored semi-quantitatively and correlated with the histological RCC subtypes. Group comparisons were calculated with the Kruskal–Wallis test. In all cases, immunoreactivity was detected only in the tumor-associated vessels and not in tumor cells. Staining intensity was the strongest in ccRCC, followed by cpRCC and pRCC. ccRCC showed the most diffuse staining pattern, followed by cpRCC and pRCC. Our results provide a rationale for PSMA-targeted theranostic approaches in ccRCC and cpRCC.

## 1. Introduction

Renal Cell Carcinoma (RCC) is the 9th most common cancer in the male population and the 14th most common form in the female population worldwide [1]. The majority of RCC cases (>60%) are detected coincidentally via ultrasound (US) or computer tomography (CT) scans performed for other indications [2]. In localized disease, radical nephrectomy is the first-line therapeutic approach, but even despite complete removal of the primary tumor, 25% to 40% of RCC patients still develop distant metastases during follow-up. In addition, 20% to 30% of RCC patients may already suffer from metastatic disease (mRCC) at the time of initial diagnosis [3,4]. Recently, the treatment of patients with mRCC has been revolutionized by the introduction of tyrosine kinase inhibitors (TKI) and immunotherapies, which show promising results in significantly prolonging the survival of mRCC patients [5]. Nevertheless, cancer cells may develop resistance mechanisms that could weaken or abrogate the therapeutic anti-cancer effects of TKIs and immune checkpoint inhibitors (ICIs) and ultimately lead to disease progression [6]. To adapt treatment strategies to such resistance mechanisms, it is important to detect disease progression as early as possible.

In contrast to partial nephrectomy, which is the preferred surgical procedure for localized renal cell carcinoma (RCC), Cytoreductive nephrectomy (CN) is commonly performed in patients with metastatic RCC (mRCC). CN aims to remove the primary tumor in the kidney and can potentially be curative if all tumor deposits are successfully removed. However, for the majority of patients with metastatic disease, CN is considered a palliative procedure, and systemic treatments, such as targeted therapies and immunotherapies, are still necessary. CN is performed with the goal of reducing tumor burden, alleviating symptoms, and potentially enhancing the effectiveness of subsequent systemic treatments. Previous studies have demonstrated improved survival benefits when CN is combined with interferon-based immunotherapy. However, recent research has challenged the role of CN in the era of targeted therapies. The emergence of novel treatments, such as tyrosine kinase inhibitors (TKIs) and immune checkpoint inhibitors (ICIs), has led to a reevaluation of the optimal use and timing of CN in the management of mRCC. The effectiveness of targeted therapies in controlling tumor growth and improving patient outcomes has raised questions about the necessity of CN in all cases of metastatic disease. Ongoing research aims to better understand the specific patient characteristics and disease factors that may influence the benefits of CN in the context of evolving treatment strategies [2].

Currently, the European Association of Urology (EAU) guideline recommends the use of contrast-enhanced CT or magnetic resonance imaging (MRI) to detect recurrent or metastatic sites in RCC patients during follow-up [2]. However, several studies have already indicated the limitations of those conventional imaging modalities in differentiating malignant from benign renal neoplasms, and consequently, the sensitivity for detecting lymph nodes or distant metastases of RCC is limited [7,8,9]. Thus, there is a need for new imaging techniques that could aid in the diagnosis and staging of RCC and facilitate the assessment of response to systemic therapy in mRCC [10].

In general, ^18^F-fluoro-2-deoxy-2-D-glucose (^18^F-FDG) for Positron Emission Tomography (PET) (^18^F-FDG PET) is widely used for detecting and monitoring tumors. However, unlike in most other malignancies, the application of ^18^F-FDG PET and hybrid PET imaging in mRCC is of limited diagnostic yield due to the low ^18^F-FDG-avidity of metastatic RCC lesions. Therefore, ^18^F-FDG PET is not recommended by practice guidelines for mRCC imaging [8].

An alternative target for radiopharmaceutical-based imaging is prostate-specific membrane antigen (PSMA), a type II integral membrane glycoprotein originally discovered on prostatic epithelium [11]. To date, PSMA PET/CT is a well-established imaging modality in patients with metastatic prostate cancer [12]. However, PSMA expression is also reported in the tumor-associated neovasculature of a variety of tumor entities, including hepatocellular carcinoma, breast cancer, and RCC [13,14]. Pilot studies with small case numbers have already investigated the clinical value of ^68^Ga-PSMA-11 PET/CT or ^18^F-PSMA-1007 PET/CT in RCC patients and suggested a potential benefit of PSMA PET/CT for staging and follow-up [15,16,17,18,19]. In addition, analogous to prostate cancer, upregulation of PSMA in the neovasculature of RCC may represent a target for potential new and innovative radiopharmaceutical treatment strategies, especially when all other therapeutic options have been exhausted [15].

Histopathologically, RCC is divided into several subtypes that differ at histomorphological and molecular levels as well as in terms of their clinical behavior. Three major histological subtypes, which together account for more than 95% of cases, are defined: Clear cell RCC (ccRCC, accounting for 80–90% of cases), papillary RCC (pRCC, 10–15%) and chromophobe RCC (cpRCC, 4–5%) [20]. A better understanding of PSMA expression in different subtypes of RCC and adjacent healthy renal tissue is crucial to assess which patients would benefit most from theranostic approaches with PSMA-targeted ligands. However, to date, only a few studies have investigated PSMA expression in RCC at the protein level [21,22,23,24]. Here, we aimed to investigate how PSMA expression is distributed in the major histological subtypes of RCC to identify those that might benefit most from a theranostic approach with PSMA ligands.

## 2. Materials and Methods

### 2.1. Tissue Samples

This retrospective study was approved by the medical ethics committee of Ludwig Maximilian University (LMU), Munich. Formalin-fixed and paraffin-embedded material from 65 patients who underwent surgery for RCC at the Department of Urology (LMU Munich) between 2011 and 2019 was collected. Tissue samples were first analyzed on hematoxylin and eosin (H&E) stained slides and classified according to the WHO Classification of Tumours, Fifth Edition. A representative section of each case was selected for analysis, on which both tumor tissue and normal kidney tissue were encountered. Tumor-associated vessels were identified on H&E and Elastica van Gieson (EvG) stain.

### 2.2. Immunohistochemical Analyses

Immunohistochemical staining for PSMA was performed on 5 µm thick formalin-fixed and paraffin-embedded (FFPE) tissue sections. Sections were pretreated with Ventana Cell Conditioner 1 Immunostainer (Ventana Medical Systems, Oro Valley, AZ, USA) for 1 h and then incubated with mouse PSMA monoclonal antibody 3E6 (1:50, Agilent Technologies, Santa Clara, CA, USA) for 32 min. Staining was performed using a Ventana BenchMark Ultra automated stainer and ultraView DAB kit (Ventana Medical Systems). Slides were counterstained with hematoxylin. Positive controls were used for quality assurance in each staining run.

The extent and intensity of PSMA-staining were evaluated semi-quantitatively as previously described [21] and correlated with histological RCC subtypes. For this purpose, the staining pattern was assessed in tumor cells, tumor-associated vessels, and adjacent normal kidney tissue. The intensity of PSMA staining was independently graded by two different observers (SL, VNB) on a scale of 1 to 3 (1 = no positive reaction or weak intensity, 2 = moderate intensity, 3 = strong intensity). For tumors with a focal staining pattern, the region with the highest staining intensity was scored. If the results differed, the scoring value was determined by discussion of all investigators. For statistical analyses, a staining intensity of 2 or 3 was considered strong, and a staining intensity of 1 was considered weak.

**Figure 1:**
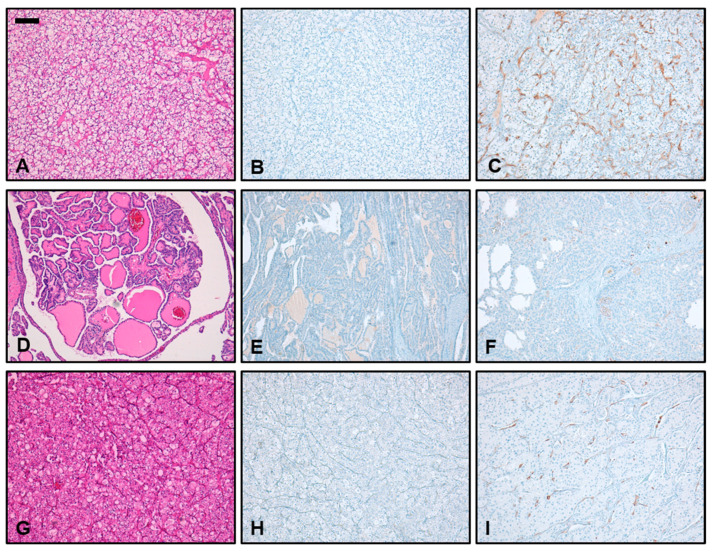
PSMA expression patterns in different histopathological subtypes of renal cell carcinoma (RCC). (**A**,**D**,**G**) Representative microphotographs of H&E-stained slides show the classic histomorphology of clear cell RCC (ccRCC, **A**), papillary RCC (pRCC, **D**), and chromophobe RCC (cpRCC, **G**). (**B**,**E**,**H**) Representative microphotographs show PSMA-stained sections of ccRCC (**B**), pRCC (**E**), and cpRCC (**H**) with minimum PSMA reactivity in the tumor-associated neovasculature. (**C**,**F**,**I**) In comparison, representative microphotographs show PSMA-stained sections of ccRCC (**C**), pRCC (**F**), and cpRCC (**I**) with maximum PSMA reactivity in the tumor-associated neovasculature. While in cRCC and cpRCC there were cases with strong and diffuse PSMA-staining (**C**,**I**), in pRCC (**F**), even maximum staining was only focal and of weak intensity (×10; scale bar 200 µm).

The distribution pattern of PSMA expression was evaluated by indicating the percentage of immunoreactive vascular structures. The pattern was considered diffuse if more than 50% of tumor-associated vessels were PSMA positive and focal if less than 50% of tumor-associated vessels were PSMA positive. Figure 1 shows representative pictures of different PSMA staining patterns.

### 2.3. Statistical Analyses

Statistical analyses were performed with IBM SPSS^®^ Statistics (version 25; SPSS, Chicago, IL, USA) and GraphPad Prism (version 9.5.0 GraphPad Software, La Jolla, CA, USA). Descriptive statistics are displayed as mean ± standard deviation (SD). A post hoc analysis from Kruskal–Wallis testing was applied to assess differences between tumor sites. Statistical significance was defined as a two-sided *p*-value < 0.05.

## 3. Results

### 3.1. Patient Characteristics

A total of 65 patients were included in the study. The median age of all patients at the time of surgery was 67 (range: 36–90) years. In total, 21/65 patients (32%) comprised ccRCC, 24/65 (37%) pRCC and 20/65 (30%) cpRCC. Demographic and tumor characteristics are shown in Table 1.

### 3.2. Immunohistochemical PSMA Expression in Non-Neoplastic Kidney Tissue

**Figure 2:**
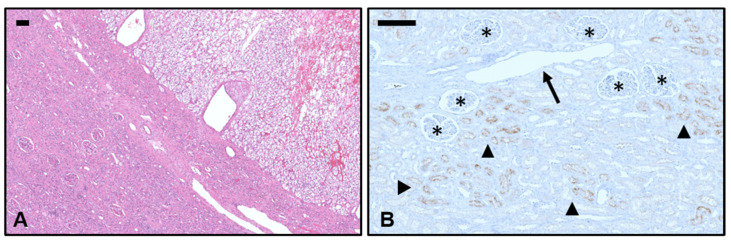
PSMA expression in non-neoplastic kidney tissue. (**A**) A representative microphotograph shows regular renal parenchyma (**lower left**) with sharply demarcated infiltration of clear cell renal cell carcinoma (**upper right**, H&E; ×10; scale bar 200 µm). (**B**) In non-neoplastic kidney tissue, PSMA expression in the epithelial cells of the tubulus system is indicated by brown color and arrowheads (PSMA; ×40; scale bar 200 µm). Nearby native renal vessels (arrow), glomeruli (asterisk), and stroma showed no immunoreactivity.

In non-neoplastic kidney tissue, PSMA was expressed in the epithelial cells of the tubulus system. Native renal vessels, glomeruli, and stroma showed no immunoreactivity (Figure 2).

### 3.3. Immunohistochemical PSMA Expression in Different Subtypes of RCC

**Figure 3:**
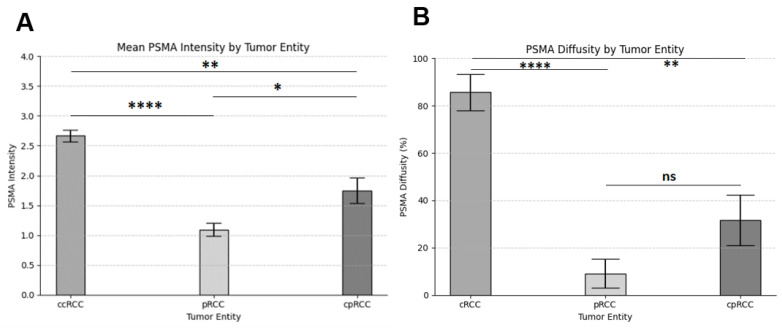
Intensity and diffusity of prostate-specific membrane antigen (PSMA) expression in different subtypes of renal cell carcinoma. (**A**) Kruskal–Wallis test shows a significant difference in PSMA intensity between clear cell renal cell carcinoma (ccRCC), papillary renal cell carcinoma (pRCC), and chromophobe renal cell carcinoma (cpRCC). In addition, cpRCC shows significantly more PSMA intensity than pRCC. (**B**) The Kruskal-Wallis test shows a significant difference in PSMA diffusity between ccRCC, pRCC, and cpRCC, respectively. The difference in PSMA diffusity between pRCC and cpRCC was not statistically significant (****, *p* < 0.0001; **, *p* < 0.01; *, *p* < 0.05).

In all cases of RCC, immunoreactivity for PSMA was detected only in the tumor-associated vessels and not in tumor cells. All (100%) ccRCC samples showed strong PSMA expression with a mean staining intensity of 2.67. In cpRCC, we found strong PSMA expression in 50% of samples, and the mean staining intensity over all samples tested was 1.75. In contrast, only 8% of pRCC showed strong PSMA expression. The mean staining intensity in pRCC was 1.08. Thus, PSMA staining intensity was strongest in ccRCC, followed by cpRCC and pRCC. Differences in PSMA intensity between ccRCC and pRCC (*p* < 0.0001), ccRCC and cpRCC (*p* < 0.01) as well as pRCC and cpRCC (*p* < 0.05) were statistically significant (Figure 3A).

The most diffuse staining pattern was observed in ccRCC, where 17/21 (81%) had a diffuse immunoreactivity for PSMA in more than 50% of tumor-associated vessels, followed by cpRCC (6/20; 30%) and pRCC (1/24, 4%). Differences in staining pattern were statistically significant between ccRCC and pRCC (*p* < 0.0001) and between ccRCC and cpRCC (*p* < 0.01). However, there was no statistical difference in PSMA diffusity between the entities of pRCC and cpRCC (Figure 3B). A summary of PSMA expression patterns in different subtypes of RCC is shown in Table 2.

## 4. Discussion

RCC is a serious malignant tumor disease that accounts for 2% of the global cancer diagnosis [1]. In the metastatic stage, the prognosis is especially poor, and the 5-year survival rate is reported to be only 12% [1]. Therapy of mRCC has been revolutionized by the development of TKIs and ICIs, to which many mRCC patients initially show good responses [5]. Unfortunately, 70% of patients develop drug resistance in the further course of the disease [6]. Sensitive, reliable, and accurate imaging modalities are needed to adapt therapy regimens to such molecular changes as quickly as possible in order to halt disease progression [10]. In addition, there is an urgent need for new therapeutic options to treat patients with therapy-resistant mRCC [25]. Analogous to prostate cancer, PSMA could act as a target for both diagnostic and therapeutic approaches. Here, we found that PSMA is expressed at different levels in the major subtypes of RCC. While native renal vessels generally did not show PSMA expression, there was a strong and diffuse PSMA expression in tumor-associated neovasculature of all ccRCC samples. In cpRCC, strong PSMA expression was observed in 50% of the cases, while in pRCC, only 8% showed strong PSMA expression. Differences in PSMA expression intensities between the different histological subtypes of RCC were statistically significant. As the immunohistochemical PSMA intensity is reported to correlate with uptake on PSMA-PET/CT, these results suggest that PSMA is suitable as a target for theranostic procedures in ccRCC and cpRCC [26].

**Figure 4:**
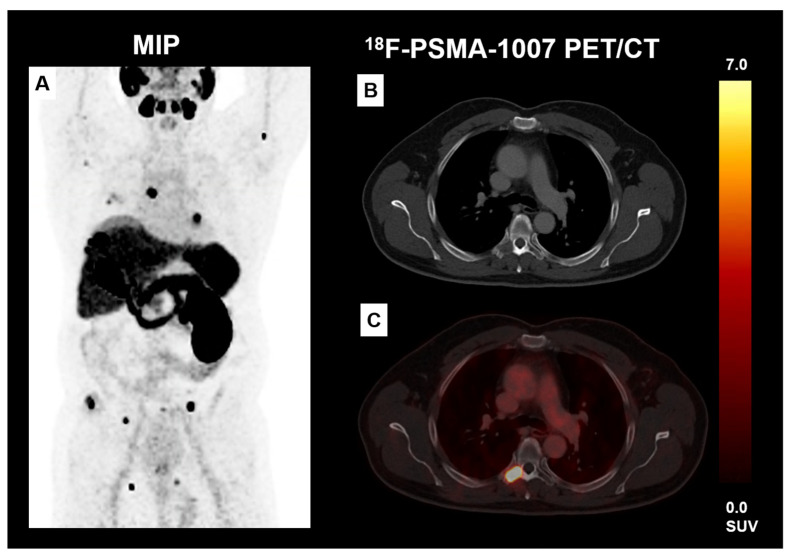
Patient with ccRCC who underwent ^18^F-PSMA-1007 PET/CT. The maximum-intensity projection (MIP) shows multifocal PSMA-positive tumoral lesions (**A**). Exemplarily PSMA-positive osteolytic lesion in the thoracic spine (**B**,**C**).

PSMA overexpression was first detected in prostate carcinomas, and PSMA-PET/CT as a form of hybrid imaging is now widely used in patients with metastatic or recurrent prostate cancer, as it shows significantly improved accuracy over conventional imaging techniques [27]. In addition, PSMA-directed radioligand therapies with lutetium-177 (^177^Lu-PSMA-617) or actinium-225 (^225^Ac-PSMA-617) are being tested in clinical trials as a therapeutic option in refractory metastatic prostate cancer [28,29,30]. Despite its name, PSMA is now known to be expressed not only in prostate carcinomas but also in the tumor-associated neovasculature of several solid tumor entities [22,23,24,31,32]. The first case series investigated the diagnostic value of ^68^Ga-PSMA-PET/CT or ^18^F-PSMA-PET/CT in RCC patients and showed promising results in ccRCC as PSMA-PET/CT could detect metastatic sites with higher sensitivity than conventional imaging methods (see exemplarily Figure 4) [8,18,33,34,35]. In non-clear cell RCC, however, only a small proportion of tumors showed uptake of PSMA-targeted radiotracer, and PSMA-PET/CT did not detect additional metastases compared to conventional imaging modalities [35]. Nevertheless, the aforementioned clinical studies on PSMA-PET/CT in RCC only investigated small case numbers, and especially non-clear cell RCCs were underrepresented [23].

Here, we demonstrated that the major RCC subtypes show significant differences in PSMA expression on protein level, with cpRCC and pRCC showing significantly less frequent and weaker PSMA immunoreactivity than ccRCC. This may be the reason for the lower diagnostic sensitivity of PSMA-PET/CT in non-clear cell RCC.

In daily practice, one major unmet need is to discriminate between malignant and benign renal lesions to avoid unnecessary biopsies or overtreatment. While we focused exclusively on malignant renal lesions in our study, preliminary research results suggest that PSMA-PET/CT could be helpful in the discrimination of benign and malignant renal neoplasms. For example, an early pioneer study by Baccala et al. reported positive PSMA staining in 76.2% of ccRCCs and only 52.6% of oncocytomas [22]. In a comparable study, 80% of ccRCC and only 30% of oncocytomas showed neovasculature with positive PSMA staining [21]. However, only a few cases were investigated, and the difference in staining intensity was not statistically significant. Nevertheless, these initial results are promising and suggest that PSMA expression may aid in the distinction between malignant and benign renal lesions. Thus, further studies are needed to investigate the PSMA expression in benign renal lesions.

Besides tumor dignity, treatment decisions in RCC patients are mainly guided by tumor stage and grade, but radiomorphologic correlates for these parameters are currently lacking. Studies have shown that PSMA expression leads to neoangiogenesis in different tumor entities, which is an important factor for tumor progression [36,37]. Consequently, PSMA expression is associated with more malignant tumor behavior, higher tumor stages, and a worse prognosis in prostate cancer, squamous cell cancer, and breast cancer [38,39,40]. In our study with RCCs, we found no difference in PSMA intensity according to clinicopathological parameters. All ccRCCs showed strong PSMA intensity regardless of stage (T1, T2, T3) or grading (G1, G2, G3). Similarly, the vast majority of pRCCs showed weak PSMA expression regardless of stage or grading, and likewise, no corresponding correlation was found in cpRCCs. Nevertheless, the significance of those results is limited due to the size of the individual subgroups. In addition, our cohort did not include T4 or G4 carcinomas. Therefore, to clarify whether PSMA-PET/CT can aid in the accurate assessment of tumor size and grading in RCCs, further studies are needed.

Another point of great clinical importance is the detection rate of metastases at the initial diagnosis of RCC. Depending on the tumor stage, different therapeutic regimens can be considered for the patient, leading to either local tumor therapy alone or systemic combination therapies with a higher risk of associated toxicities [2]. In mRCC patients undergoing TKI or ICI therapy, reliable imaging methods are critical for response assessment or detection of disease progression. In this setting, PSMA-PET/CT could be a highly promising method because this molecular imaging method seems to be able to detect disease progression earlier than conventional imaging [15]. Our results suggest that the use of PSMA-PET/CT could be particularly useful in patients with ccRCC and, to a lesser extent, in cpRCC.

PSMA is a transmembrane protein and consists of 19 intracellular, 24 transmembrane, and 707 extracellular amino acids. The protein is responsible for various different enzymatic activities, although its precise function is still not fully understood. To date, it is known that PSMA contributes to tumor progression in a number of ways. For example, PSMA acts as a folate hydrolase, breaks down polyglutamate folate chains, and enables the uptake of monoglutamate folate [41]. The increased cellular folate uptake is an essential component for enhanced nucleic acid synthesis by dysregulated tumor cells [40,42]. Consequently, PSMA-positive prostate cancer cells were shown to have a greater invasive potential [42], and breast cancer cells with downregulated PSMA expression exhibited decreased cell proliferation and migration, suggesting that PSMA contributes to carcinogenesis and metastasis [43]. In addition, PSMA plays a key role in the neoangiogenesis of solid tumors. PSMA inhibition, knockdown, or deficiency led to abrogation of angiogenesis [44]. Against this background, it is readily explained why therapy directed against PSMA can halt tumor progression in patients with metastatic prostate cancer.

Similar to this, PSMA radioligand therapy could also be an option for end-stage mRCC patients for whom there are no other treatment options [8,11]. As with all therapeutic modalities, a trade-off between cancer control and therapy-associated side effects is necessary. Studies to date have indicated a low risk of nephrotoxicity with the use of ^177^Lu-PSMA-617 for the therapy of hormone-refractory metastatic prostate cancer [45,46]. Nevertheless, nephrotoxicity and tubulointerstitial nephritis are possible side effects, and renal function should be monitored closely during PSMA therapy [47]. As a possible pathogenetic cause, we found PSMA expression in the epithelial cells of the (proximal) tubule system, which is consistent with previous findings in the literature [48]. In addition, PSMA expression has been described in salivary glands, the brain, and small intestinal tissue, which is why treating physicians should be alert for side effects in these organs when using PSMA-labeled radioligands [31,48].

Given potential side effects and high therapy costs, it is critical to identify those patients who would benefit most from PSMA theranostics. Therefore, a profound knowledge of PSMA expression in normal kidney tissue and histopathological subtypes of RCC is important. The expression of PSMA in RCCs has been investigated by immunohistochemistry in some studies, mainly including ccRCC and pRCC [21,22,23,24]. Here, in agreement with our results, PSMA expression was found to be vigorous in the majority of ccRCCs, whereas PSMA expression was rarely detected in pRCCs. Moreover, a significant association between high PSMA expression and overall survival was demonstrated in ccRCC patients [24]. Our study was able to confirm and reproduce the pattern and tendency in PSMA expression of previous studies. However, those studies used internal domain-binding antibodies, which limits the clinical applications because antibody-bound radioligands exert their therapeutic effects mainly through extracellular antigens [22,36,41].

It is also possible that different PSMA-targeting antibodies recognize and bind different splicing forms of PSMA [14]. Therefore, in our study, we used an extracellular epitope PSMA targeting antibody (3E6).

Data on PSMA expression in cpRCC have also been reported, but the sample sizes were considerably smaller, and thus, it is not clear to date whether cpRCC patients may also benefit from PSMA theranostics. However, especially in metastatic cpRCC, PSMA radioligand therapy is of high clinical interest because there are currently no established treatment options for this rare form of RCC [49]. In our study, we detected PSMA expression in 50% of cpRCC cases, demonstrating a rationale for PSMA theranostics in this subtype of RCC, too.

PSMA-targeted endoradiotherapy could be used to enhance the response to immunotherapy via the abscopal effect in RCC due to its high immunoresponsiveness. The abscopal effect is a phenomenon where localized radiation therapy can trigger an immune response throughout the body [50,51]. Additionally, the cross-fire effect and the radiation-induced bystander effect (RIBE) have been discussed as potential mechanisms for the efficacy of therapeutic radioligand therapy. The cross-fire effect is achieved by particle-induced destruction of multiple cells in the neighborhood of a tracer accumulating cell. This mechanism helps to compensate for the heterogeneity seen in malignant tumors. Correspondingly, RIBE is a phenomenon in which cells that are not directly exposed to ionizing radiation behave as if they have been exposed [52]. These mechanisms are of particular interest for potential theranostic applications, as PSMA is found mostly on the neovasculature of renal neoplasms in contrast to prostate tumors, where it is mainly expressed by the carcinoma cells.

However, clinical trials involving α- or β-emitter-radiolabeled PSMA-targeted ligand therapy on RCC have yet to be conducted.

Our study is limited because we focused on the three major histopathological subtypes of RCC and other rare RCC entities, and benign renal neoplasms, such as oncocytoma, were not included. Future studies should be performed to investigate PSMA expression in those other RCC subtypes. Also, the intensity of the PSMA expression could be correlated with the respective T stage of the RCC entity. In addition, in vivo and in vitro autoradiography binding studies are needed to accurately determine the binding affinity of PSMA-targeted radioligands in RCC samples. Further (multicenter) studies could also focus on the change of PSMA expression under systemic therapy and investigate possible differences in PSMA expression between tissue samples from primary tumors and metastases, which may provide further insights into the role of PSMA in tumor progression and metastasis.

## 5. Conclusions

Based on our immunohistochemical study, we found statistically significant differences in PSMA expression patterns within the tumor neovasculature of ccRCC, cpRCC, and pRCC. The observed variations in PSMA expression underscore the potential for PSMA-targeted theranostic approaches. Given that ccRCC is the most prevalent subtype of RCC, the ability to selectively target PSMA could have a substantial clinical impact. PSMA-target therapies—including radioligand therapy—have already shown significant potential in the treatment of prostate cancer.

While our findings are promising, further research is required to translate these results into practical applications. Further prospective (multicenter) studies are needed to validate our findings across larger and more diverse patient populations. These studies can help assess the diagnostic and therapeutic potential of PSMA radioligands with greater precision and provide essential data for clinical implementation.

## Figures and Tables

**Table 1:** Clinical and pathological characteristics.

n = 65	ccRCC (n = 21)		pRCC (n = 24)		cpRCC (n = 20)	
	PSMA Low (n = 0)	PSMA Strong (n = 21)	PSMA Low (n = 22)	PSMA Strong (n = 2)	PSMA Low (n = 10)	PSMA Strong (n = 10)
Median age (years, range)	--	71 (37–82)	68 (47–82)	78 (73–83)	69 (38–90)	61 (36–72)
Sex						
Male, n (%)	--	15 (71)	21 (95)	2 (100)	6 (60)	7 (70)
Female, n (%)	--	6 (29)	1 (5)	0 (0)	4 (40)	3 (30)
T stage						
T1, n (%)	--	16 (76)	14 (64)	1 (50)	6 (60)	7 (70)
T2, n (%)	--	1 (5)	2 (9)	1 (50)	3 (30)	2 (20)
T3, n (%)	--	4 (19)	6 (27)	0 (0)	1 (10)	1 (1)
T4, n (%)	--	0 (0)	0 (0)	0 (0)	0 (0)	0 (0)
Lymph node status						
NX, n (%)	--	21 (100)	20 (91)	1 (50)	10 (100)	9 (90)
N0, n (%)	--	0 (0)	1 (5)	0 (0)	0 (0)	1 (10)
N1, n (%)	--	0 (0)	1 (5)	1 (50)	0 (0)	0 (0)
Lymphovascular invasion						
L0, n (%)	--	21 (100)	21 (95)	1 (50)	10 (100)	10 (100)
L1, n (%)	--	0 (0)	1 (5)	1 (50)	0 (0)	0 (0)
Vascular invasion						
V0, n (%)	--	19 (90)	21 (95)	1 (50)	10 (100)	9 (90)
V1, n (%)	--	2 (10)	1 (5)	1 (50)	0 (0)	1 (10)
Perineural invasion						
Pn0, n (%)	--	21 (100)	22 (100)	2 (100)	10 (100)	10 (100)
Pn1, n (%)	--	0 (0)	0 (0)	0 (0)	0 (0)	0 (0)
Distant metastasis						
MX, n (%)		1 (5)	3 (14)	1 (50)	10 (100)	1 (10)
M0, n (%)	--	20 (95)	18 (82)	1 (50)	0 (0)	9 (90)
M1, n (%)	--	0 (0)	1 (5)	0 (0)	0 (0)	0 (0)
Residual status						
RX, n (%)	--	2 (10)	0 (0)	0 (0)	0 (0)	0 (0)
R0, n (%)	--	19 (90)	21 (95)	2 (100)	9 (90)	10 (100)
R1, n (%)	--	0 (0)	1 (5)	0 (0)	1 (10)	0 (0)
Grading						
G1	--	5 (24)	6 (27)	1 (50)	NA	NA
G2	--	11 (52)	10 (45)	0 (0)	NA	NA
G3	--	5 (24)	4 (18)	1 (50)	NA	NA
G4	--	0 (0)	1 (5)	0 (0)	NA	NA

ccRCC, clear cell renal cell carcinoma; pRCC, papillary renal cell carcinoma; cpRCC, chromophobe renal cell carcinoma; NA, not applicable.

**Table 2:** PSMA expression in renal cell carcinoma.

Tumor Subtype	ccRCC (n = 21)		cpRCC (n = 20)		pRCC (n = 24)	
	Focal (%)	Diffuse (%)	Focal (%)	Diffuse (%)	Focal (%)	Diffuse (%)
Staining Extent	4 (19%)	17 (81%)	14 (70%)	6 (30%)	23 (91%)	1 (4%)
	Weak (%)	Strong (%)	Weak (%)	Strong (%)	Weak (%)	Strong (%)
Staining Intensity	0 (0%)	21 (100%)	10 (50%)	10 (50%)	22 (92%)	2 (8%)
Mean Staining Intensity	2.67 (±0.47)		1.75 (±0.94)		1.08 (±0.49)	

ccRCC, clear cell renal cell carcinoma; pRCC, papillary renal cell carcinoma; cpRCC, chromophobe renal cell carcinoma.

## Data Availability

Primary data are available for bonafide researchers upon request from the corresponding author.
